# Supplementary material for: SNX18 regulates ATG9A trafficking from recycling endosomes by recruiting Dynamin‐2
Source: EMBO Rep. 2018 Feb 7;19(4):e44837. doi: 10.15252/embr.201744837 (PMC5891424; doi:10.15252/embr.201744837)
Supplement: Supplementary file 1 — Expanded View Figures PDF [file EMBR-19-e44837-s001.pdf]

Expanded View Figures

Figure EV1. SNX18 regulation of autophagic proteins.

A HEK293A cells were fixed and immunostained with antibodies against endogenous SNX18 and ATG9A before confocal microscope analysis. Scale bar = 10 μm.
B HEK293A control or SNX18 KO cells were transfected with GFP-LC3B. Seventeen hours post-transfection, the cells were starved or not for 2 h in EBSS ± 100 nM BafA1, before fixation and analysis by high-throughput imaging (Zeiss AxioObserver, 20× objective). Scale bar = 10 μm.
C The number of GFP-LC3 spots observed in (A) was quantified from 40 fields of view (minimum 2,000 cells) per condition and quantitation of punctate objects using CellProfiler software (mean ± SEM from $n = 3$ independent experiments). Significance was determined by two-way ANOVA and Bonferroni post-tests where $^{*}P < 0.05$.
D HEK293A control or SNX18 KO cells were starved or not in EBSS for 4 h ± 100 nM BafA1, before cell lysis and immunoblotting of p62. Actin was used as loading control.
E The p62 levels observed in (C) were quantified and normalised to fed within each cell line. The graph shows (mean ± SEM, $n = 3$), analysis by two-way ANOVA and Bonferroni post-test determined no significance between cell lines.
F The level of mitophagy was determined by stable expression of a mitochondrial localised mCherry-GFP tag. Mitophagy was induced by treatment of cells with 1 mM DFP for 24 h prior to fixation and high-throughput analysis with a Zeiss AxioObserver widefield microscope (20×) to monitor for the formation of red only puncta. The number of red only puncta was determined by CellProfiler software from 30 fields of view and normalised to control cells with no treatment from $n = 2$ experiments. Each point represents a single replicate from a minimum of 1,000 cells per treatment.
G The levels of ATG9 observed in Fig 1G were quantified relative to actin and normalised to fed control cells (mean ± SEM, $n = 3$). Analysis by two-way ANOVA and Bonferroni post-test determined no significance between cell lines.
H Gene expression of SQSTM1, ATG9A, ATG16L1 and SNX9 was quantified by qPCR in HEK293A control or SNX18 KO cells. The graph shows the mean relative gene expression normalised to control cells from three independent experiments (mean ± SEM, $n = 3$). Analysis by two-way ANOVA and Bonferroni post-test determined no significance difference of targets between cell lines.
I The levels of TfR observed in Fig 1G were quantified relative to actin and normalised to control fed cells (mean ± SEM, $n = 3$). Significance was determined by two-way ANOVA and Bonferroni post-tests where $^{*}P < 0.05$.
J HEK293A control or SNX18 KO cells were transfected with control siRNA or siRNA targeting ULK1 for 72 h, and cells were then starved or not for 2 h in EBSS ± 100 nM BafA1 before cell lysis and Western blot analysis. Actin was used as a loading control.
K LC3 lipidation (LC3-II) from (I) was quantified, and the graph shows the average level of LC3-II relative to actin and normalised to Ctrl fed (mean ± SEM), $n = 5$. Significance was determined by two-way ANOVA and Bonferroni post-test where $^{***}P < 0.001$.

© 2018 The Authors

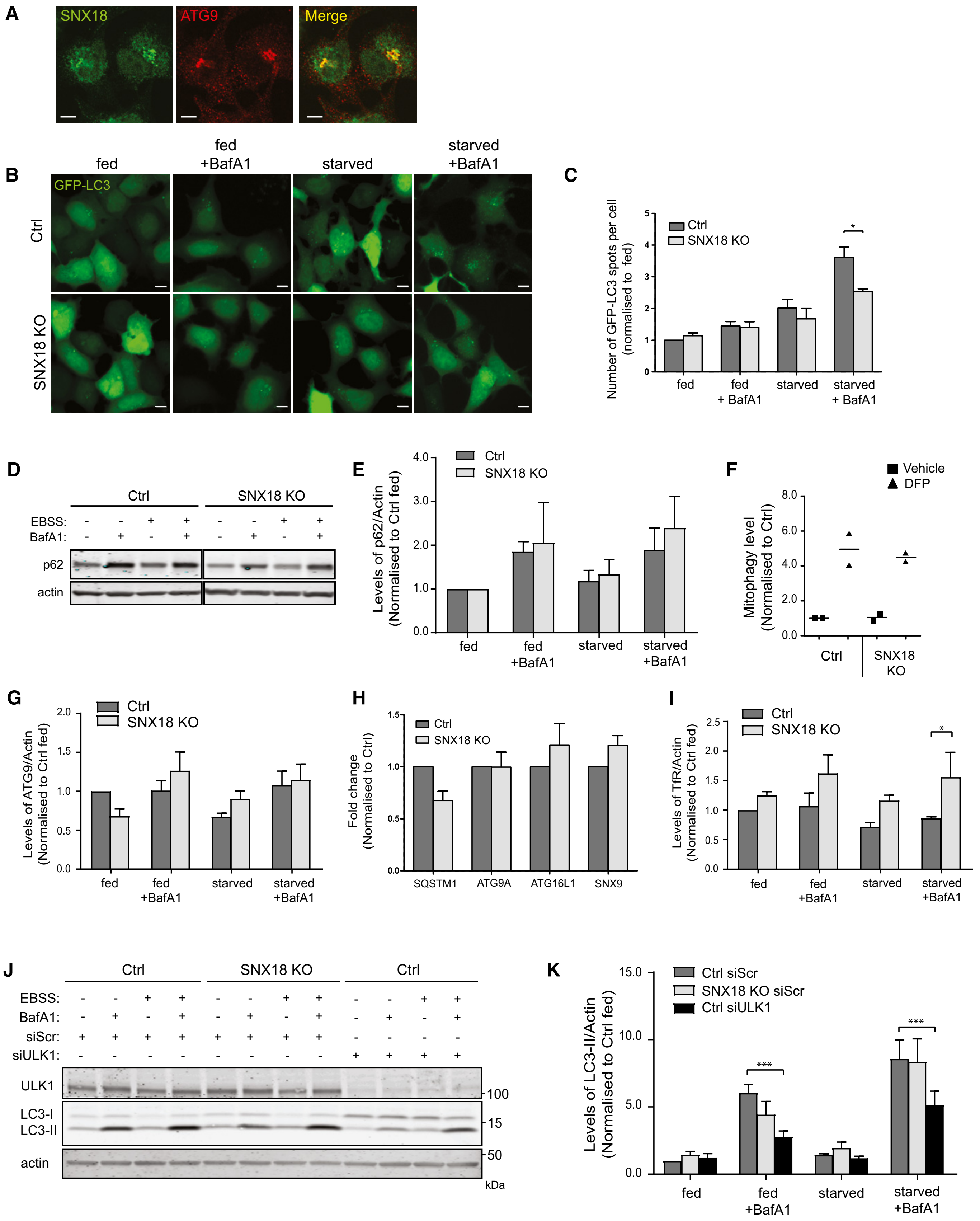

Figure EV1.

 © 2018 The Authors

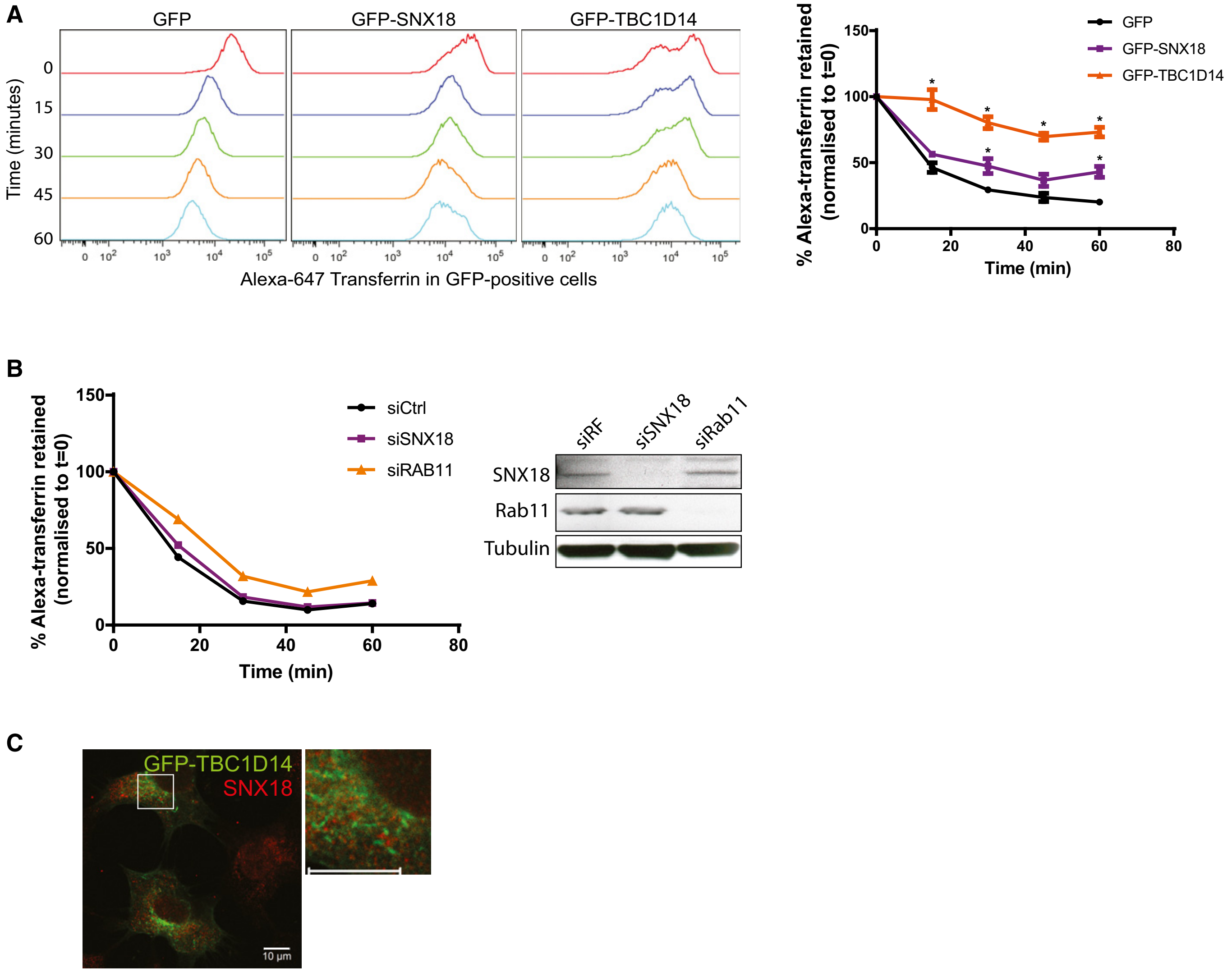

Figure EV2. The effect of SNX18 and TBC1D14 on recycling endosome function.

A HEK293A cells were transfected with GFP, GFP-SNX18 or GFP-TBC1D14. The cells were incubated with Alexa-647 transferrin for 15 min in complete medium, followed by a chase for the indicated periods. At the end of each time point, the cells were trypsinised, fixed and analysed by flow cytometry. The line graph shows the percentage of retained Alexa-647 transferrin in GFP-positive cells, normalised to $t = 0$ (mean ± SEM, $n = 3$). *$P < 0.05$ by Student's t-test.

B HEK293A cells were transfected with control, SNX18 or RAB11 siRNA for 3 days, then incubated with Alexa-647 transferrin, treated and analysed as in (A). Knockdown efficiency was verified by immunoblotting using the indicated antibodies.

C HEK293A cells were transfected with GFP-TBC1D14, fixed and immunostained with an antibody against endogenous SNX18, followed by confocal microscope analysis. Scale bar = 10 µm.

© 2018 The Authors

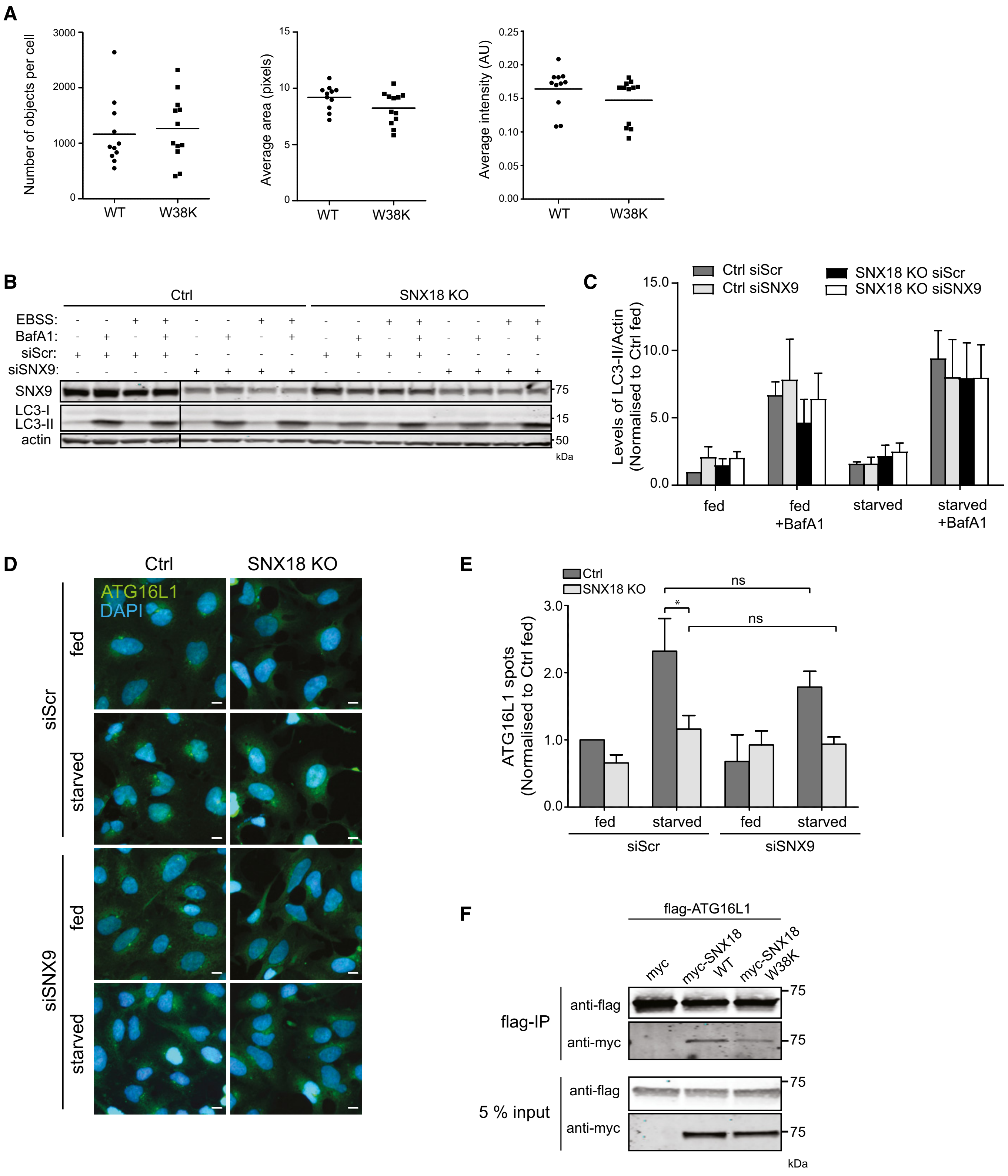

Figure EV3.

 © 2018 The Authors

Figure EV3. SNX9 knockdown does not exacerbate SNX18 KO phenotype.

A The number of Dynamin puncta observed in Fig 3B and D was quantified using CellProfiler software. The graphs show average object number, object area and intensity plotted per field of view (11 per condition) from $n = 1$ (bar represents mean value).

B HEK293A control or SNX18 KO cells were transfected with control siRNA or siRNA targeting SNX9 for 72 h, and cells were then starved or not for 2 h in EBSS ± 100 nM BafA1 before cell lysis and immunoblotting using the indicated antibodies. Actin was used as a loading control.

C LC3-II levels were quantified from each condition relative to actin and normalised to fed control cells (mean ± SEM, $n = 3$). Analysis by two-way ANOVA and Bonferroni post-test determined no significance difference between cell lines or treatment with siRNA.

D HEK293A control or SNX18 KO cells were transfected with either control siRNA or SNX9 siRNA for 72 h before starvation in EBSS for 2 h followed by fixation and immunostaining against endogenous ATG16L1 before analysis with high-throughput imaging (Zeiss AxioObserver, 20× objective). Scale bar = 10 μm.

E The number of ATG16L1 spots observed in (D) was quantified using CellProfiler software from 25 fields of view per condition and normalised to Ctrl fed siScr (mean ± SEM, $n = 3$). Significance was determined between cell lines by two-way ANOVA and Bonferroni post-test where $^*P < 0.05$. Comparison of siScr starved to siSNX9 starved by one-way ANOVA and Bonferroni post-test was not significant (ns) in either Ctrl or SNX18 KO cells.

F HEK293A cells were cotransfected with myc control, myc-SNX18 WT or myc-SNX18 W38K together with flag-ATG16L1 before cell lysis. The lysates were incubated with magnetic anti-flag microbeads before immunoblotting of the cell lysate (input) and the immunoprecipitate (flag-IP) with antibodies against flag and myc.

© 2018 The Authors
